# Improving the Robustness of Electromyogram-Pattern Recognition for Prosthetic Control by a Postprocessing Strategy

**DOI:** 10.3389/fnbot.2017.00051

**Published:** 2017-09-27

**Authors:** Xu Zhang, Xiangxin Li, Oluwarotimi Williams Samuel, Zhen Huang, Peng Fang, Guanglin Li

**Affiliations:** ^1^CAS Key Laboratory of Human-Machine Intelligence-Synergy Systems, Shenzhen Institutes of Advanced Technology, Chinese Academy of Sciences, Shenzhen, China; ^2^Department of Biomedical Engineering, Southern University of Science and Technology, Shenzhen, China; ^3^Department of Biomedical Engineering, University of Connecticut, Storrs, CT, United States; ^4^Shenzhen Institutes of Advanced Technology, University of Chinese Academy of Sciences, Shenzhen, China; ^5^Department of Rehabilitation Medicine, Panyu Center Hospital, Guangzhou, China

**Keywords:** pattern recognition, electromyogram, myoelectric prosthesis, motion onset detection, postprocessing, robustness, amputee, rehabilitation robotics

## Abstract

Electromyogram (EMG) contains rich information for motion decoding. As one of its major applications, EMG-pattern recognition (PR)-based control of prostheses has been proposed and investigated in the field of rehabilitation robotics for decades. These prostheses can offer a higher level of dexterity compared to the commercially available ones. However, limited progress has been made toward clinical application of EMG-PR-based prostheses, due to their unsatisfactory robustness against various interferences during daily use. These interferences may lead to misclassifications of motion intentions, which damage the control performance of EMG-PR-based prostheses. A number of studies have applied methods that undergo a postprocessing stage to determine the current motion outputs, based on previous outputs or other information, which have proved effective in reducing erroneous outputs. In this study, we proposed a postprocessing strategy that locks the outputs during the constant contraction to block out occasional misclassifications, upon detecting the motion onset using a threshold. The strategy was investigated using three different motion onset detectors, namely mean absolute value, Teager–Kaiser energy operator, or mechanomyogram (MMG). Our results indicate that the proposed strategy could suppress erroneous outputs, during rest and constant contractions in particular. In addition, with MMG as the motion onset detector, the strategy was found to produce the most significant improvement in the performance, reducing the total errors up to around 50% (from 22.9 to 11.5%) in comparison to the original classification output in the online test, and it is the most robust against threshold value changes. We speculate that motion onset detectors that are both smooth and responsive would further enhance the efficacy of the proposed postprocessing strategy, which would facilitate the clinical application of EMG-PR-based prosthetic control.

## Introduction

Hands are important parts of the human body, which are used to perform various dexterous daily actions in an intuitive manner. Complete or partial loss of upper limb would greatly affect the life activities of amputees. In an attempt to rehabilitate upper-limb amputees, many research efforts have been made toward the development of efficient upper-limb prosthetic devices worldwide. Electromyogram (EMG) contains rich information which could be used to decode the motion intentions. It has been shown that even complex movements can be identified from EMG using a dynamic recurrent neural network approach (Dipietro et al., 2003; Cheron et al., 2007), which manifest the possibility of manipulating complex prostheses through the EMG signal. On the other hand, the progress of prosthetic control studies has been relatively slow. An early proposed prosthetic control methodology, based on the threshold detection of surface EMG measured from residual arms, has been commercially applied for a long time. It determines the motion outputs upon detection of the activity from the corresponding muscles. However, this kind of control method is cumbersome and counterintuitive (Kuiken et al., 2009; Li et al., 2010; Scheme and Englehart, 2011); in addition, the performance of the conventional EMG control method is limited by a number of factors such as surface EMG crosstalk and variability in signal amplitude (Farina et al., 2014). An alternative and more promising approach is EMG pattern-recognition (EMG-PR)-based prosthetic control method, which could enable amputees to intuitively conduct prosthetic devices with multiple DoFs (Oskoei and Hu, 2007). This approach usually works by extracting a number of features from the segmented EMG signals in real time and then using a trained classifier to map the EMG features onto a corresponding target motion class that serves as an input to the prosthetic controller. The EMG-PR approach has been widely proposed and investigated for decades and achieved a decent classification accuracy of over 95% from offline analysis (Englehart and Hudgins, 2003; Li et al., 2016a), but its clinical applications is still limited. The most critical issue might be the unsatisfactory robustness and reliability of EMG-PR-based movement identifications, which are caused by some inevasible interferences in the practical uses such as electrode shifts (Hargrove et al., 2008; Young et al., 2011), muscle fatigue (Wan et al., 2010), and change in limb positions (Scheme et al., 2010; Fougner et al., 2011; Geng et al., 2012).

These interferences may lead to misclassifications that constantly exist and vary during all muscle contraction phases. For example, during a dynamic contraction, a transition is made between the rest phase and an active muscle contraction, the corresponding EMG portion is thus unstable, which leads to a majority of misclassifications in the EMG-PR scheme, as reported in a recent study (Lorrain et al., 2011). The same study addressed the problem by including the dynamic EMG portion into the training set, which proved effective in improving the classification accuracy during dynamic contractions. Besides, other contraction phases are subject to the interferences as well. For instance, during the rest phase when there is no intended limb motion, external noise may induce disturbances in the EMG signal, which could lead to misclassifications. In addition, during a constant contraction, a certain motion class is held constant, while muscle fatigue and force variations may destabilize the EMG signal, and in turn reduce the classification accuracy. To diminish erroneous outputs during all these contraction phases, there have been a number of approaches that improve the performance of different stages of the myoelectric control scheme, namely signal preprocessing or filtering (Zhou et al., 2007; Hargrove et al., 2009; Phinyomark et al., 2009; Hofmann et al., 2016), data windowing (Smith et al., 2011), feature extraction (Phinyomark et al., 2009, 2012; Rafiee et al., 2011; Veer and Sharma, 2016; Samuel et al., 2017), and classification (Oskoei and Hu, 2007; Adewuyi et al., 2016). In addition, a few strategies that add a postprocessing stage after the original classification output (OCO) have been proposed to suppress misclassifications, which are referred to as postprocessing strategies in this study. For myoelectric control, the postprocessing strategy is used to determine whether the current classification output is directly sent to the prosthetic controller or otherwise, through a postprocessing step before sent to the controller. In this way, the strategy may reduce erroneous outputs by blocking the potential misclassifications. The majority vote is among the earliest of such strategies that improve the overall classification accuracy and robustness, where the current motion output is determined by the past three or more outputs (Englehart and Hudgins, 2003). However, the majority vote may not be able to suppress two or more consecutive misclassifications. Recently, another postprocessing strategy has been proposed where the current output is determined by the maximum likelihood estimation from a classifier and the forearm muscle activity (Amsüss et al., 2014). Nevertheless, due to factors such as muscle fatigue, the muscle activity and the EMG signals may change over time during constant contractions, which may affect the probability estimation and lead to unwanted rejection or change of motion outputs, thus reducing the efficacy of the strategy. Hence the existing methods might still be insufficient, particularly for tasks which require continuously correct motion outputs over a relatively long contraction period.

This study proposed a postprocessing strategy that stabilizes motion outputs during the constant contraction upon a threshold-based motion onset detection process, to improve the robustness of myoelectric control especially during the rest phase and constant contractions. Three commonly adopted motion onset detectors, namely the mean absolute value (MAV) (Englehart and Hudgins, 2003), Teager–Kaiser energy operator (TKE) (Li et al., 2007), and mechanomyogram (MMG) (Orizio, 2004), were adopted and examined individually for the proposed strategy in order to test and analyze their performance. To evaluate the online performance of the strategy, several metrics such as the overall error rates, motion-specific error rates and switching rates, were chosen and calculated with the data recorded during an online task in a virtual environment. Furthermore, this study provided a comparison among different motion onset detectors, and proposed the characteristics of motion onset detectors that are key to further enhancing the efficacy of the proposed strategy.

## Materials and Methods

### Participants and Equipment

A total of 10 subjects including eight able-bodied individuals and two male transradial amputees were recruited in this study. The able-bodied subjects include six males and two females, aged between 20 and 33 years old, and they had certain experience (ranging from a few months to 2 years) in myoelectric control experiments. One amputee was 27 years old with a right arm amputation for about 8 years and another aged 32 years old with a left arm amputation for about 10 years. In addition, the two amputees have been using a body-powered prosthesis for >5 years. All the recruited subjects were given written informed consent and they provided permission for the publication of their photographs and data for scientific and educational purposes. The protocol of this study was approved by the ethics committee of Institutional Review Board of Shenzhen Institutes of Advanced Technology, Chinese Academy of Sciences, China.

**Figure 1:**
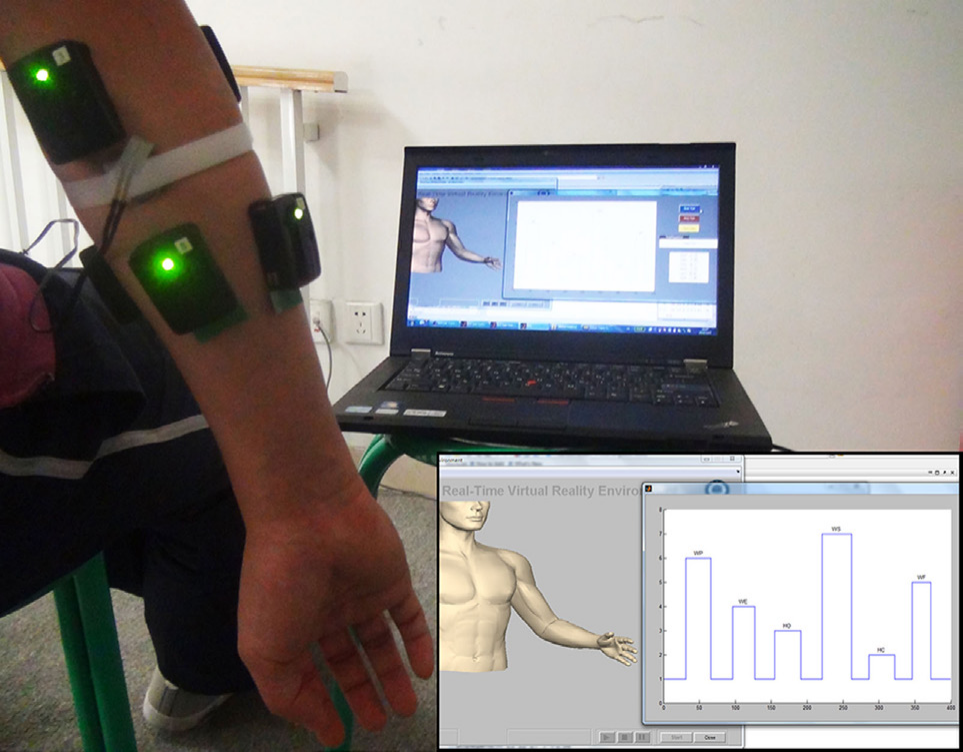
Placement of EMG and force-sensing resistor (FSR) sensors on a representative subject. The inset on the lower right corner shows the online testing interface, which will be mentioned in the Section “Online Testing Protocol.”

In the study, a wireless physiological signal acquisition system (Trigno Wireless EMG System, Delsys, Inc., Boston, MA, USA) was used to record the surface EMG signals. In addition, a single-zone force-sensing resistor (FSR) sensor (FSR 402, Interlink Electronics, Inc., CA, USA) was connected to the EMG system to simultaneously record the MMG signals. As representatively shown in Figure 1, each participant was instructed to sit on an armless chair in a comfortable manner. The FSR sensor was firstly mounted on the skin surface between the *palmaris longus* and *flexor carpi ulnaris* of her/his dominant forearm, and then fixed firmly with an inelastic bandage to avoid any relative displacement between the FSR sensor and the skin as well as the bandage. Subsequently, four bipolar surface EMG electrodes were placed evenly around the forearm skin right below the bandage, roughly covering the *extensor digitorum*, the *flexor digitorum*, the *extensor carpi*, and the *flexor carpi*, respectively. Two other electrodes were placed right above the bandage on the *extensor* and the *flexor*, respectively.

### Offline Classifier Training and Testing

Seven motion classes, namely hand close (HC), hand open (HO), wrist extension (WE), wrist flexion (WF), wrist pronation (WP), wrist supination (WS), and no-movement (NM), were included in the study because they are commonly performed during daily tasks, and represent the degrees of freedom in three dimensions (HC/HO, WE/WF, and WP/WS), which have also been used in previous studies (Lorrain et al., 2011; Connan et al., 2016; Li et al., 2016b). Following a video prompt, all the subjects performed one after another of the above motion classes in a fixed sequence by holding the corresponding muscle contraction for 4 s with a comfortable and consistent force level, and resting for 3 s before the next contraction. The motion execution sequence was repeated four times. The EMG data during each contraction were labeled as the corresponding motion class and later used as the training set. After 2 min rest, the above procedure was repeated once and the EMG data were used as the testing set. During the whole signal acquisition process, the MMG was simultaneously recorded along with the EMG. Both signals were sampled at 1,000 Hz.

The recorded EMG data were segmented using a sliding window with a length of 150 ms and an increment of 100 ms (50 ms overlapping) (Smith et al., 2011), which resulted in 159 windows for each motion class in either the training or the testing set. From each analysis window, four commonly used time domain features, namely MAV, number of zero crossings, waveform length, and slope sign changes, were extracted from all six channels of EMG data for each motion class in both the training and the testing sets (Hudgins et al., 1993; Scheme and Englehart, 2011; Li et al., 2016b). Each motion class in the training or the testing datasets is thus a 159 × 24 matrix. A classifier based on the linear discriminant analysis (Englehart and Hudgins, 2003; Adewuyi et al., 2016) was trained and tested with fivefold cross validation. The classification error rate was employed to quantify the offline classification performance of the trained classifier, which is defined as the percentage of the number of misclassifications over the number of total classifications in the testing set.

### Online Testing

#### Online Processing Scheme

**Figure 2:**
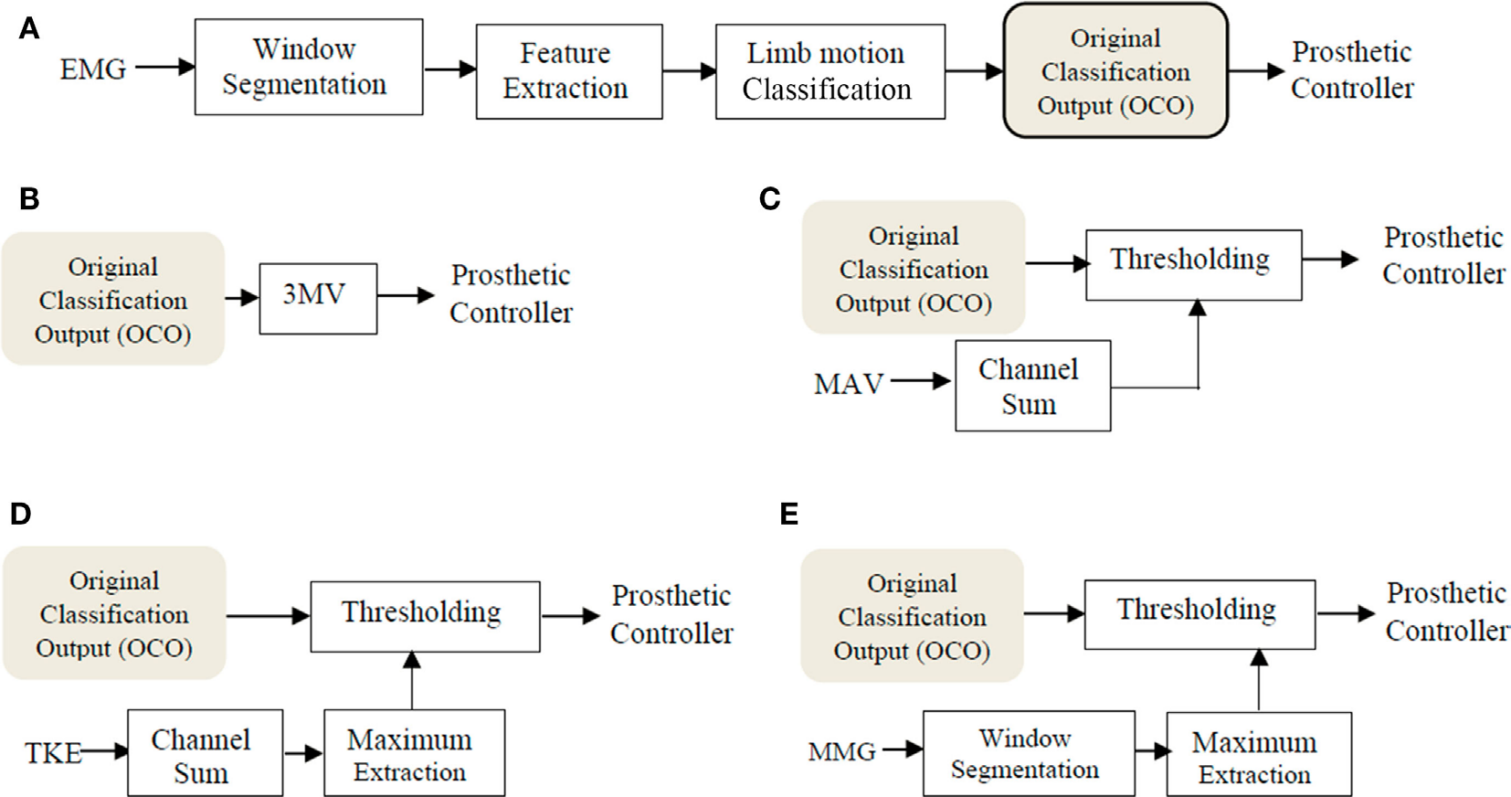
The online processing schemes for five scenarios. **(A)** Original classification output (OCO): The conventional electromyogram-pattern recognition (EMG-PR) scheme, where the original classification output was directly sent to the prosthetic controller. **(B)** 3MV: a 3-point majority vote was applied to the original classification output before sending it to the prosthetic controller. **(C)** Mean absolute value (MAV): the proposed postprocessing strategy based on MAV was adopted, where the MAV signal was obtained from the feature extraction step in the conventional EMG-PR scheme, and then implemented in the thresholding scheme after summing across all EMG channels. **(D)** Teager–Kaiser energy operator (TKE): the proposed postprocessing strategy based on TKE is adopted, where the TKE was computed for each EMG data point, summed across all EMG channels, and then implemented in the thresholding scheme after extracting the maximum in each window. **(E)** Mechanomyogram (MMG): the proposed postprocessing strategy based on MMG is adopted, where the MMG signal was acquired from the force-sensing resistor (FSR) sensor, and the maximal value of MMG was extracted in each window and then implemented in the thresholding scheme.

The schematic diagrams in Figure 2 show the general processing schemes corresponding to five different scenarios labeled from (A) to (E). Scenarios (A) and (B) are conventional schemes which have been adopted by a number of studies before, and are described as follows:

(A) Original classification output: the OCO was obtained from the conventional EMG-PR scheme as proposed by several previous studies (Hudgins et al., 1993; Englehart and Hudgins, 2003; Adewuyi et al., 2016). In this scheme, the OCO is directly sent to the prosthetic controller as shown in Figure 2A.
(B) 3-Point majority vote (3MV): a 3MV step is added to the OCO as previously proposed (Englehart and Hudgins, 2003), which has been the most commonly adopted postprocessing strategy so far.

The three Scenarios (C) to (E) with the proposed postprocessing strategy are shown in Figures 2C–E. Three different motion onset detectors namely MAV, TKE, and MMG were implemented by Scenarios (C) to (E), respectively. Each of the three scenarios added a common thresholding scheme before sending the OCO to the prosthetic controller, so as to stabilize the motion outputs. However, the derivation and threshold setting for the three motion onset detectors were different, which are described, respectively, as below.

(C) Mean absolute value: the MAV feature extracted from EMG data points in each window is commonly used as a measure to estimate the contraction force for proportional control in prostheses (Hudgins et al., 1993) and has also been proposed as a motion onset detector to suppress actuation of the prosthetic system when below a certain threshold (Englehart and Hudgins, 2003). The sum of the six MAV values corresponding to the six channels of EMG data was computed for each analysis window as shown in Eq. 1, and both the onset and the offset thresholds of the EMG burst were set as 3 positive standard deviations of the baseline based on Eq. 2:
  (1) $$\text{ext}{\text{MAV}}_{i}=\sum_{j=1}^{6}{\text{MAV}}_{i,j},$$
  (2) $$\text{onThMAV}=\text{off ThMAV}=\bar{u}(n)+3\text{SD}\left[u(n)\right],$$
(D) Here MAV*_i,j_* stands for the original MAV of the *j*th electrode channel and extMAV*_i_* is the extracted MAV from the *i*th analysis window; onThMAV and offThMAV represent the onset and offset thresholds for MAV, respectively, *n* is the number of window and *u*(*n*) is the baseline value of MAV. TKE: TKE has been adopted to detect limb motion onset from EMG signals with high signal to noise ratio (Li et al., 2007; Solnik et al., 2008). In this study, the maximum TKE value was extracted from each analysis window after summing across all the EMG channels, and the TKE value of every data point except the first and last was computed based on the mathematical expression shown in Eq. 3:
  (3) $${\text{TKE}}_{i,j}(k)=x^{2}(k)-x\left(k-1\right)x\left(k+1\right),$$
  (4) $${\text{extTKE}}_{i}=\text{max}\left\{\sum_{j=1}^{6}{\text{TKE}}_{i,j}(k)\right\},$$
  (5) $$\text{onThTKE}=\text{off ThTKE}=\bar{v}(k)+3\text{SD}\left[v(k)\right],$$
  where TKE*_i,j_* (*k*) stands for the original TKE of the *j*th electrode channel of the *k*th point in each window, and extTKE*_i_* is the extracted TKE from the *i*th analysis window; onThTKE and offThTKE represent the onset and offset thresholds for TKE, respectively, *x*(*k*) is the EMG value of the *k*th point in each window and *v*(*k*) is the baseline value of TKE. Further, the highest TKE value for all channels in each analysis window was extracted based on Eq. 4. Then, the onset and offset thresholds of EMG burst for TKE were set in a manner similar to that of MAV as shown in Eq. 5.
(E) MMG: MMG, also named as FMG (force-myogram), measures muscle vibrations, and can be obtained by piezoelectric contact sensors, accelerometers, microphones and so on (Madeleine et al., 2001; Orizio, 2004; Silva et al., 2005; Castellini and Ravindra, 2014; Li et al., 2016c), besides FSR as adopted by this study and recent researches (Ravindra and Castellini, 2014; Connan et al., 2016). The amplitude of MMG has strong correlation with the muscle contraction force applied and even a little change in force strength would result to a noticeable change in its amplitude; besides, its relatively high correlation with the timing of motion onset and offset makes it a good candidate for motion onset detection (Wininger et al., 2008). From the segmented MMG data, the maximum value among the data points in each analysis window was extracted to represent the MMG value for that window by using Eq. 6:
  (6) $${\text{extMMG}}_{i}=\text{max}\left\{{\text{MMG}}_{i}(k)\right\},$$
  (7) $$\text{onThMMG}=\frac{1}{3}\text{min}\left\{\text{IPV}(l,m)\right\},$$
  (8) $${\text{off ThMMG}}_{l}=\frac{2}{3}\text{min}\{{\text{PA}}_{l}(m)\},$$
  where MMG*_i_* (*k*) stands for the original MMG of the *k*th point in each window, and extMMG*_i_* is the extracted MMG from the *i*th analysis window; onThMMG represents the onset threshold for MMG, while offThMMG*_l_* is the offset threshold for motion class *l* for MMG. IPV(*l, m*) and PA*_l_*(*m*) are the initial peaking value and plateau amplitude of motion class *l* in trial *m*, respectively. To determine the onset MMG threshold value for the EMG burst, the contraction in the training set across all motion classes that produced the lowest initial peaking amplitude was selected and one third of the amplitude was designated as the onset threshold as shown in Eq. 7. Also, the offset MMG threshold value for the EMG burst was obtained for individual motion class, where two thirds of the plateau’s amplitude during constant contractions was set as the offset threshold for that specific motion class as shown in Eq. 8.

**Figure 3:**
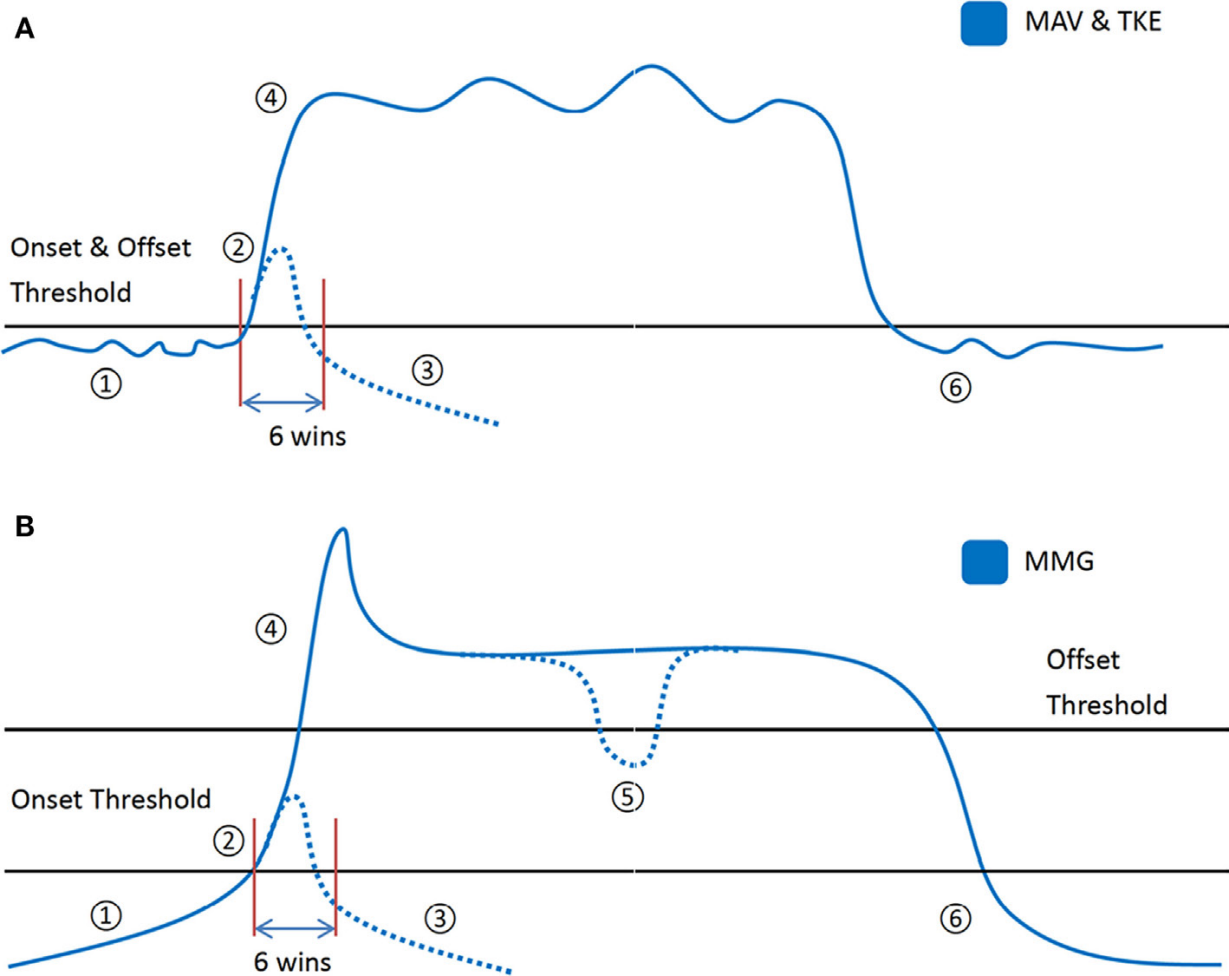
The thresholding procedure based on **(A)** mean absolute value (MAV) or Teager–Kaiser energy operator (TKE), where there were five states in a muscle contraction labeled as number 1–4 and 6, and **(B)** mechanomyogram (MMG), where the six states in a contraction are labeled as number 1–6.

In Scenarios (C) to (E), the OCO is processed by a thresholding scheme once the motion onset detectors and their respective threshold values are obtained. The application procedures of the thresholding scheme for the proposed strategy involve transitions among multiple states, which are conceptually illustrated in Figure 3 and also described as follows:

(1) State 1 (rest stage): when the amplitude of the motion onset detector is below the onset threshold, the output is set to NM.
(2) State 2 (decision stage): when the motion onset detector’s amplitude exceeds the onset threshold value, the output remains unchanged as the OCO for next six active windows (each window has a length of 150 ms with an increment of 100 ms as previously stated). If the amplitude of the motion onset detector drops below the onset threshold during the six windows, the state shifts to State 3. Otherwise, the majority motion class in the six active (non-NM) outputs is defined as the *decision output*, and State 4 ensues thereafter.
(3) State 3 (rest stage): when the amplitude of the motion onset detector drops below the onset threshold, the output is set to NM and it goes back to the case of State 1.
(4) State 4 (locking stage): the *decision output* obtained in State 3 would be set as the constant output throughout the contraction period. The contraction period lasts until the amplitude of the motion onset detector drops below the offset threshold as set previously. When the offset threshold is set higher than the onset threshold, as in the case of MMG (Figure 3B), State 5 ensues if the amplitude the motion onset detector is still above the onset threshold. Otherwise, the direct dropping of the amplitude below the onset threshold leads to State 6 (for MAV and TKE in the study, as shown in Figure 3A).
(5) State 5 (unlocking stage): when the motion onset detector’s amplitude is between the onset and offset threshold values (Figure 3B), the output motion class is reset to NM. If the amplitude continues to drop below the onset threshold, it leads to State 6. Otherwise, if the amplitude rises above the offset threshold again within three windows, the state returns to State 4 where the *decision output* is kept the same as previously. However, if the unlocking stage lasts longer, it goes back to State 2 where the *decision output* will be determined again.
(6) State 6 (rest stage): when the amplitude of the motion onset detector drops below the onset threshold, the output is reset to NM and the offset threshold is reset as null, which returns to the case of State 1. It should be noted that there is a minimal duration of resting phase required for performing a different motion class after completion of a previous one.

#### Online Testing Protocol

**Figure 4:**
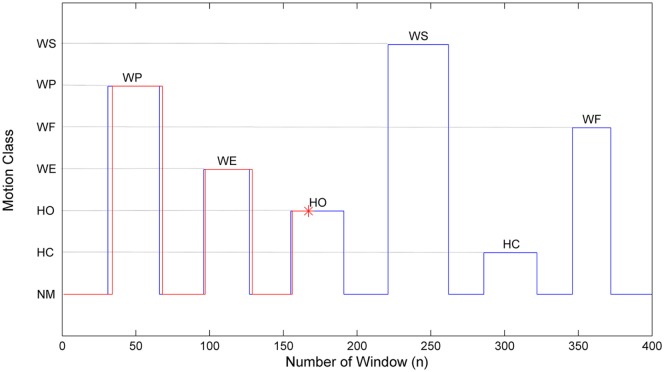
The online test interface. The blue line represents the predesigned motion sequence while the red line represents the actual motion output of a subject in the mechanomyogram scenario. The asterisk indicates the current window of motion execution.

The online testing session consisted of a designated sequence of limb motions as WP-WE-HO-WS-HC-WF, and each motion lasted for around 2 s, followed by a rest also around 2 s, as shown in Figure 4. The blue line shows the designated start and end points of each motion, while the red line shows the real-time motion execution by the subject with the MMG-based strategy, and the asterisk shows the current window. Subjects might start a muscle contraction earlier or later than the designated time, depending on their response duration. A trial is successfully completed when the subject performs the required motions as closely to the predesigned sequence as possible. Four consecutive trials were performed by each subject, and the outputs from all the five different scenarios (A–E in the Section “Online Processing Scheme”) were obtained simultaneously. The values of MAV, TKE, and MMG in each window were recorded for later use.

#### Online Performance Analysis

The online performance of all the motion outputs was evaluated using six metrics, including total error rate (TER), motion error rate (MER), rest error rate (RER), motion-specific total error rate (MSTER), motion-specific active error rate (MSAER), and motion-specific switching rate (MSSR), as defined in Eqs 9–14.

(9) $$\text{TER}=\frac{\text{Number of errors during the whole online task}}{\text{Length of the designated sequence}},$$

(10) $$\text{MER}=\frac{\text{Number of errors during contractions}}{\text{Length of the designated motion sequence}},$$

(11) $$\text{RER}=\frac{\text{Number of errors during rest}}{\text{Length of the designated rest sequence}},$$

(12) $$\text{MSTER}=\frac{\text{Number of errors during a specific motion}}{\text{Length of a specific motion in the designated sequence}},$$

(13) $$\text{MSAER}=\frac{\text{Number of non-NM errors during a specific motion}}{\text{Length of a specific motion in the designated sequence}},$$

(14) $$\text{MSSR}=\frac{\begin{array}{c} \text{Number of output changes to a wrong}\\ \text{non-NM motion classes during a specific motion} \end{array}}{\text{Length of a specific motion in the designated sequence}},$$

Total error rate, MER, and RER are overall metrics which represent the error rate for different periods in a complete online task. TER can be derived from MER and RER given the respective lengths of contraction and rest periods. While the motion errors constitute a major part of the total errors, the rest errors, which would lead to unwanted activation of prostheses, also affect the robustness of prosthetic control. In addition, MSTER, MSAER, and MSSR can reflect the online performance for each specific motion class. The active error (represented by MSAER), as similarly proposed in a previous study (Amsüss et al., 2014), is usually considered as a major factor that would limit the myoelectric control performance, because misclassifications into any other active motion classes would affect the user experience to a greater extent than those into NM (which are later written as non-active errors). Moreover, the switching rate (represented by MSSR) is proposed to indicate how many times the output changes to a wrong active (non-NM) motion class from the rest or the correct motion class during a constant contraction.

### Sensitivity Analysis of Motion Onset Detectors

The sensitivity of the motion onset detectors (MAV, TKE, and MMG) was analyzed and compared based on their performances over a certain range of onset threshold values, which were represented by the motion error and the rest error as defined before. For MAV and TKE, the threshold ranges were set between 0.1 and 10 times of their respective initial values, whereas for MMG, the onset threshold value range was set from half to twice of its initial value, and the offset threshold value was set as previously described and kept unchanged. The online test results corresponding to each threshold value were reconstructed by imposing the new threshold value on the recorded MAV, TKE, and MMG in each window during the whole online task. The motion errors and the rest errors were obtained, respectively, from the reconstructed results for each threshold value. In this way, different motion onset detectors were compared in terms of their respective sensitivity to threshold value changes.

## Results

### Offline Classification Performance

The offline classification accuracy of each motion class is shown in the confusion matrix in Table 1. A total classification accuracy of 96.3 ± 2.3% was obtained across all the 10 subjects through fivefold cross-validation. It could be observed from the confusion matrix that the classification performance of each motion class was relatively satisfactory, although WS had the lowest classification accuracy at 92.3%.

**Table 1:** Averaged offline classification accuracy (%) of each motion class shown in a confusion matrix.

Total classification accuracy: 96.3 ± 2.3							
Predicted Actual	HC	HO	WE	WF	WP	WS	NM
HC	94.7	1.1		0.1	1.1	0.9	2.2
HO	0.1	97.6			0.2	1.0	1.1
WE			98.1		0.2	1.1	0.6
WF	0.2			97.5		1.6	0.7
WP		0.1	0.2		95.4	0.9	3.4
WS	0.7	0.4	0.1	0.5	2.3	92.3	3.8
NM		0.2			1.0	0.1	98.7

*The numbers in each column are the percentage of the actual motion class (corresponding to the leftmost column) classified into the predicted motion class (corresponding to the uppermost column). The results along the main diagonal show the percentage of correct classifications or accuracy, and their mean is the total classification accuracy of the classifier as shown on the top of the table. The other elements show the percentage of incorrect classifications or error rate. Empty cells correspond to an error rate less than 0.05%*.

### Evaluation of Online Test Performance

**Figure 5:**
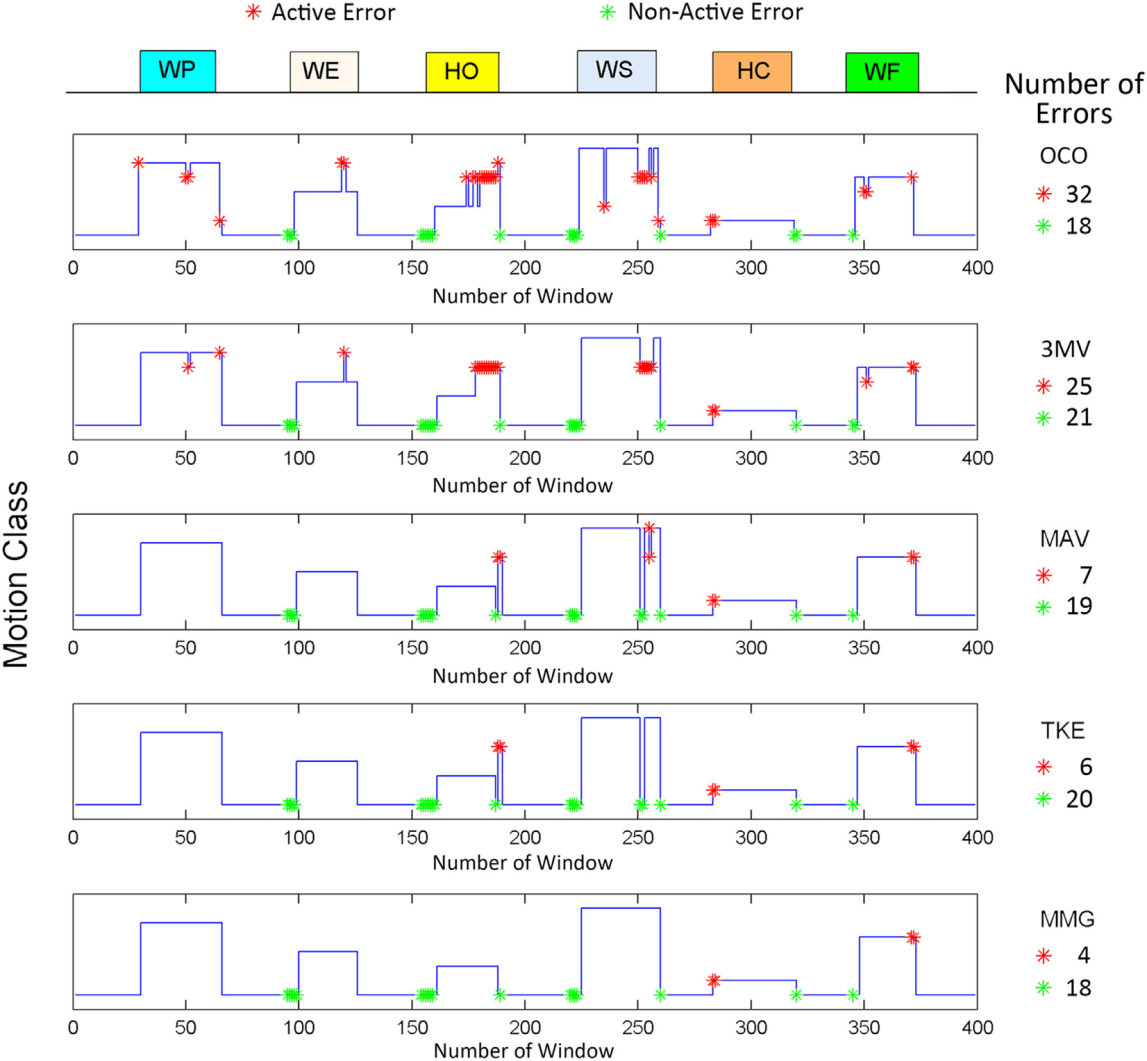
A sample result of the online test for five different schemes. In each plot, the horizontal axis represents the number of window while the vertical axis shows the actual motion class performed by the subject, in the same way as shown in Figure 4. The arrow on the top with bars of different colors shows the designated motion classes. The red and green asterisks represent the active and the non-active errors, respectively, with corresponding error numbers in the whole output sequences.

A typical result for a trial of the online test is shown in Figure 5, where those outputs that were different from the designated motion sequence are labeled by color asterisks. The red asterisks indicate the active errors (misclassifications into an erroneous active motion class), and the green asterisks indicate the non-active errors (misclassifications into NM), which have been defined above in Online performance analysis section. As shown in Figure 5, 32 active errors occurred in the OCO sequence, and even in the 3MV scenario where a 3MV is additionally applied to OCO, the number of active errors was still as high as 25. It could be also observed that most of the active errors occurred at the later period of muscle contractions, especially for HO and WS, which were likely induced by muscle fatigue; while the non-active errors usually appear around the motion onsets and offsets, which are believed to be induced by the response delay of the subjects. Upon applying the proposed strategy based on the three motion onset detectors, the number non-active errors remained roughly unchanged; however, the active errors were greatly reduced to as low as 7, 6, and 4, respectively. This implies that the proposed strategy could help stabilize motion outputs during active muscle contractions.

Note that we observed that either MAV or TKE was inapplicable to the thresholding scheme for two able-bodied subjects during the execution of certain motion classes, which will be mentioned in the Discussion part. The problem did not exist in the case of MMG, though. Therefore, the results of the two subjects were excluded in the following analyses.

#### Overall Metrics

**Figure 6:**
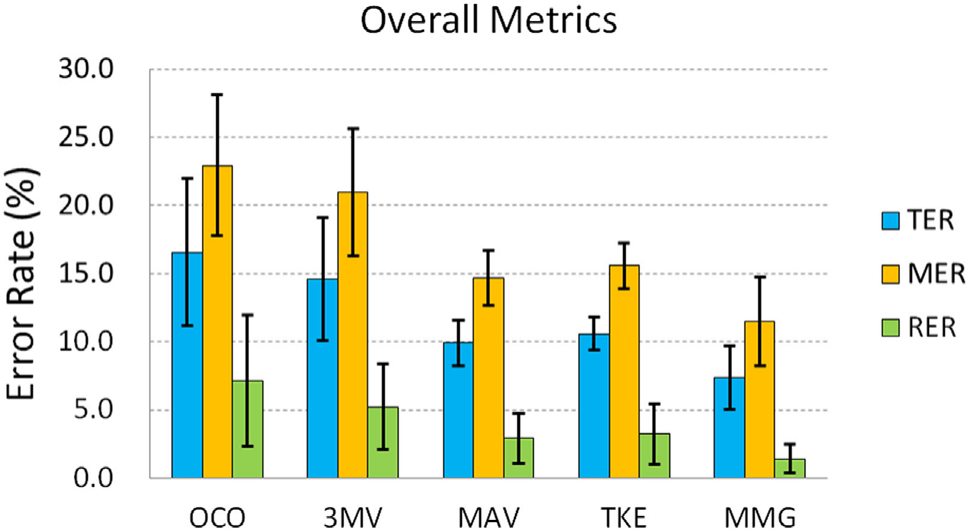
The overall metrics of total error rate (TER, in blue), motion error rate (MER, in orange), and rest error rate (RER, in green). Error bars stand for standard deviations.

A comparison among all the five scenarios based on TER, MER, and RER was performed. The averaged results for the three overall metrics are presented in Figure 6. By dividing TER into MER and RER, the classification error rates during the muscle contractions and the rest phases could be analyzed individually. As Figure 6 shows, both MER and RER follow basically the same trend as TER: OCO had the highest of all the three error rates among all the five scenarios, followed by the 3MV; in contrast, these error rates in the case of MAV, TKE, and MMG were much lower. Specifically, with respect to MER, both OCO and 3MV were around 20% (22.9 and 21.0%, respectively), while the average MER values for MAV, TKE, and MMG were all below 16%, and MMG reached the lowest value at only 11.5%. Then for RER, the proposed strategy reduced its average values from 7.2% in OCO to below 3.2%, and the lowest RER value was achieved by MMG again at merely 1.4 ± 1.0%. These results suggest that the overall error indices were suppressed by the proposed strategy, during both muscle contractions and rest.

**Figure 7:**
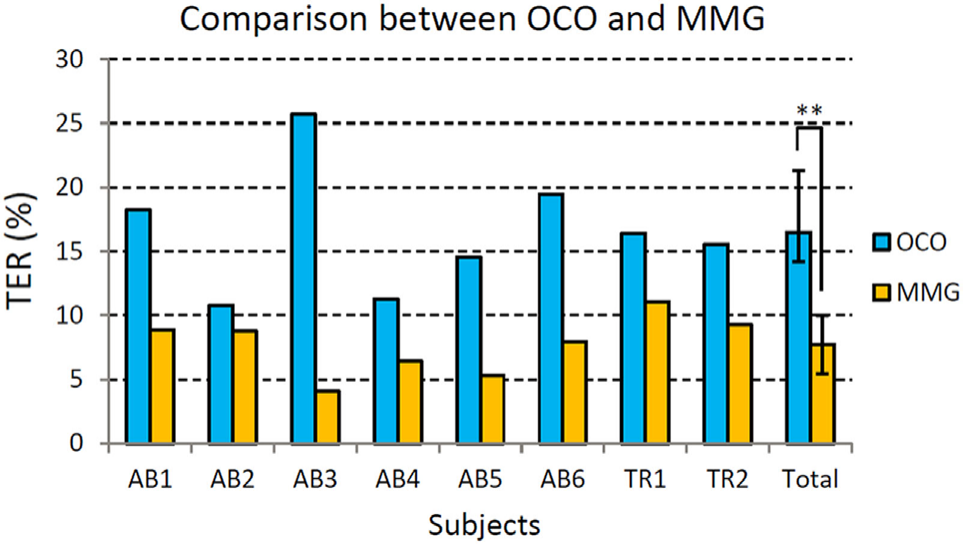
Comparison of total error rate (TER) for each individual subject between original classification output (OCO, in gray) and the motion output with mechanomyogram (MMG) thresholding (in blue). AB stands for the able-bodied subjects, and TR represents the transradial amputees. Error bars stand for standard deviations. (**In average, the TER of MMG is significantly lower than that of OCO, *p* < 0.01.)

To investigate deeper into how the improvement with the proposed strategy was achieved on each subject, we specifically plotted the TER of OCO and MMG for each subject in Figure 7 as a representative comparison. From Figure 7, it can be observed that there is no much difference between the performance of the healthy subjects and the amputees. However, each subject showed a lower TER of MMG compared with OCO. In total, there is a significant difference (based on paired *t*-test) between the TER of OCO and MMG (*p* < 0.01), which shows that the proposed strategy significantly reduced the motion output errors, and thus improved the online test performance of each subject.

#### Motion-Specific Metrics

**Figure 8:**
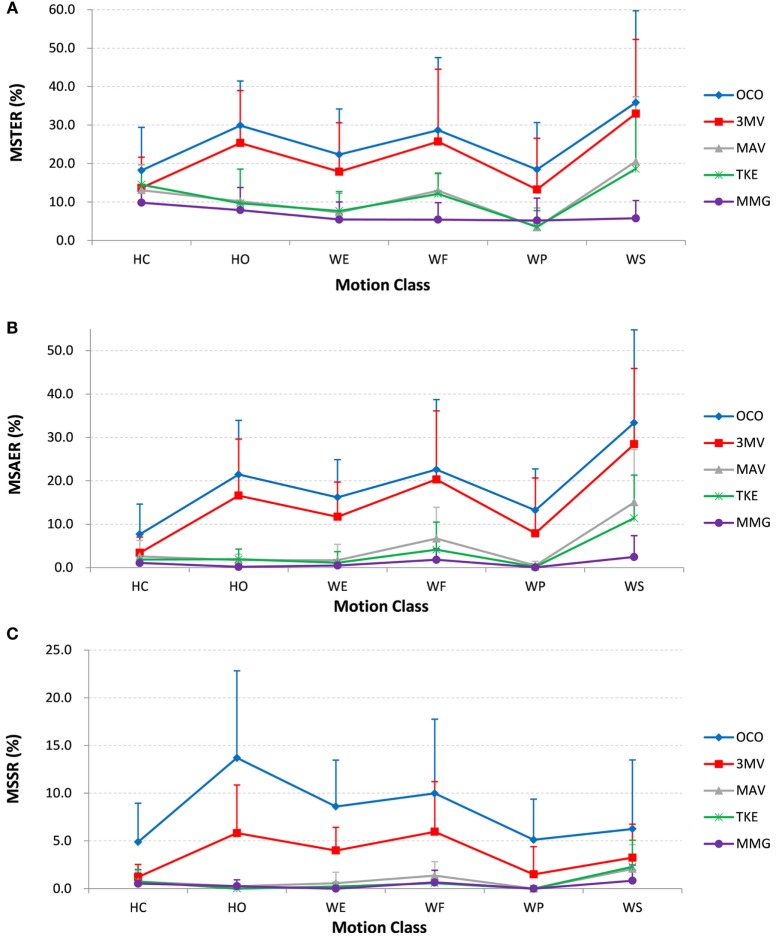
The online performance by motion-specific metrics: **(A)** motion-specific total error rate (MSTER), **(B)** motion-specific active error rate (MSAER), and **(C)** motion-specific switching rate (MSSR). Each point on the chart represents the average value across all subjects, and the single-sided error bar shows the standard deviation.

A next comparison of the online task performance among the five scenarios was based on the three motion-specific metrics namely MSTER, MSAER, and MSSR. The averaged results across all the subjects, as well as their standard deviations, are shown in Figure 8. It can be seen in Figures 8A,B that the results of MSTER for all the scenarios follow a similar trend to that of MSAER, despite that the MSTER values were generally higher. As earlier explained for the example result, active errors (MSAER) are more useful for analysis of the online performance than non-active errors, whereas MSTER counts both. With respect to MSAER, OCO had values between 7.7 and 22.6% for five of the six active motion classes, and for WS was as high as 33.4%; in addition, as shown by the error bars the standard deviation of OCO was above 6.9% for each motion class, and reached as high as 21.4% for WS, indicating that the online performance of the control scheme was highly unstable. After postprocessed by a 3MV as in the 3MV scenario, the averaged MSAER value and the standard deviation for each motion class were reduced by no more than 5.3% (WP) and 3.9% (WS) compared with OCO, which implies that the use of a 3MV strategy led to only a slight improvement. In contrast, the proposed strategy in all the three scenarios reduced the MSAER to below 6.7% with the standard deviation no more than 7.2% for five of the six motion classes except for WS, which was also reduced to no more than 15.1% with a standard deviation of 12.2%. In particular, MMG reached the lowest MSAER values for all the motion classes among the other motion onset detectors, which were at most 2.5% (WS), with a standard deviation of 4.9%. These results indicate that the erroneous motion outputs were largely and consistently reduced for each motion class.

Furthermore, as shown in Figure 8C, the MSSR values of OCO were between 4.9 and 13.7%, and 3MV had MSSR values between 1.2 and 6.0% for all the motion classes; while MAV, TKE, and MMG all achieved average MSSR values lower than 1.4% for each motion class. Especially for HO, the MSSR was 13.7% for OCO and 5.8% for 3MV, whereas the proposed strategy nearly eliminated the MSSR (all below 0.3%) with all the three motion onset detectors. Besides, the standard deviation of MSSR for OCO across each motion class was between 4.1 and 9.1%, which was reduced to between 1.3 and 5.3%, but was further reduced to no more than 3.0% for all motion classes after applying the proposed method. It shows that the unwanted motion output changes during each motion class were suppressed by the proposed strategy.

### Comparison among the Motion Onset Detectors

**Figure 9:**
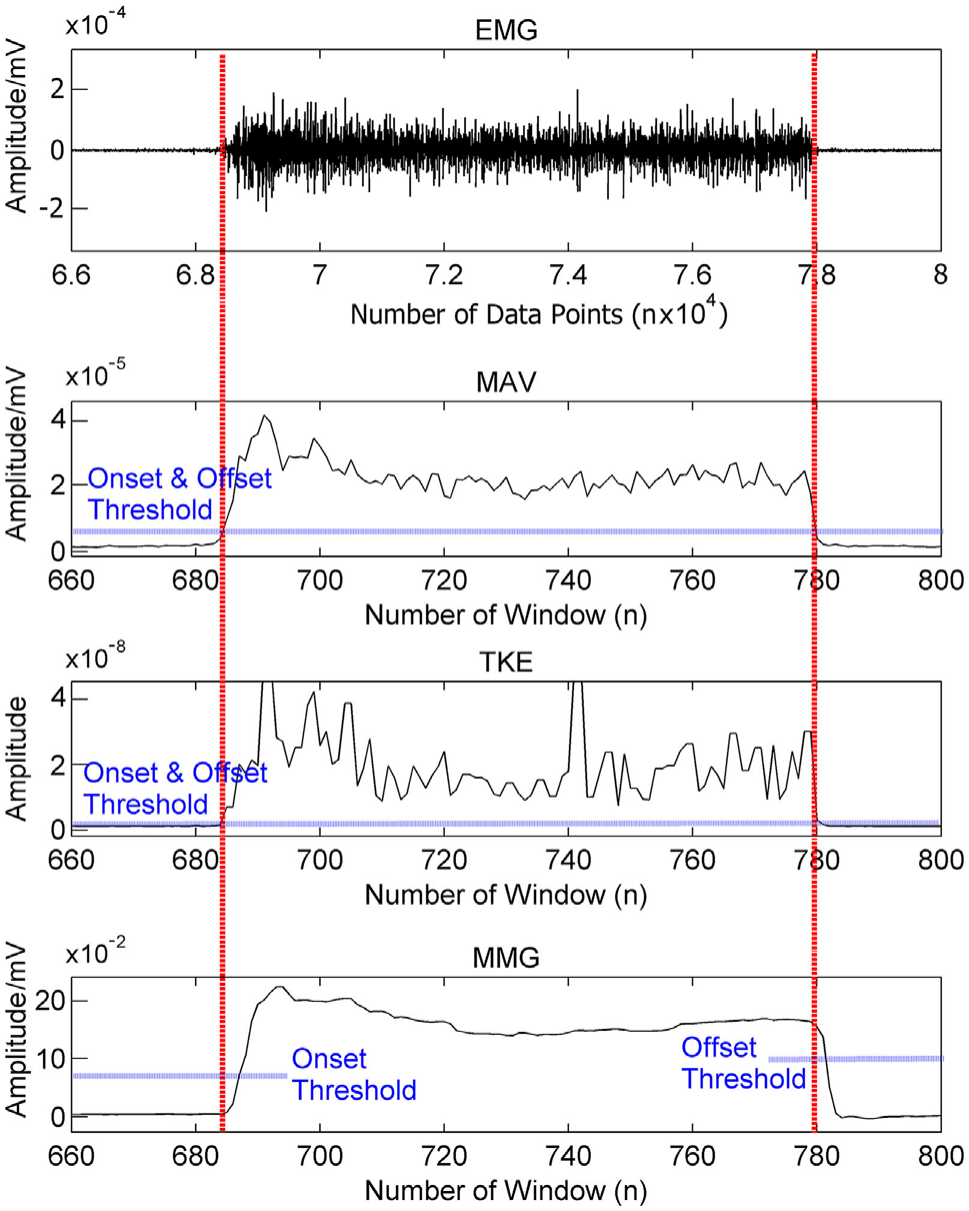
Waveforms of the original EMG signal and the three corresponding motion onset detectors obtained from a typical muscle contraction. The vertical axis of each plot shows the amplitude range of the signal.

The waveforms of the original EMG and the three corresponding motion onset detectors obtained from a typical muscle contraction are shown in Figure 9. Larger fluctuations were observed in TKE than MAV during the constant contraction, but TKE had much smaller perturbations during the rest period, implying its better ability to resist noise. In comparison, MMG produced the smoothest waveform among other motion onset detectors, but it also showed a larger delay both at the onset and offset of the contraction.

**Figure 10:**
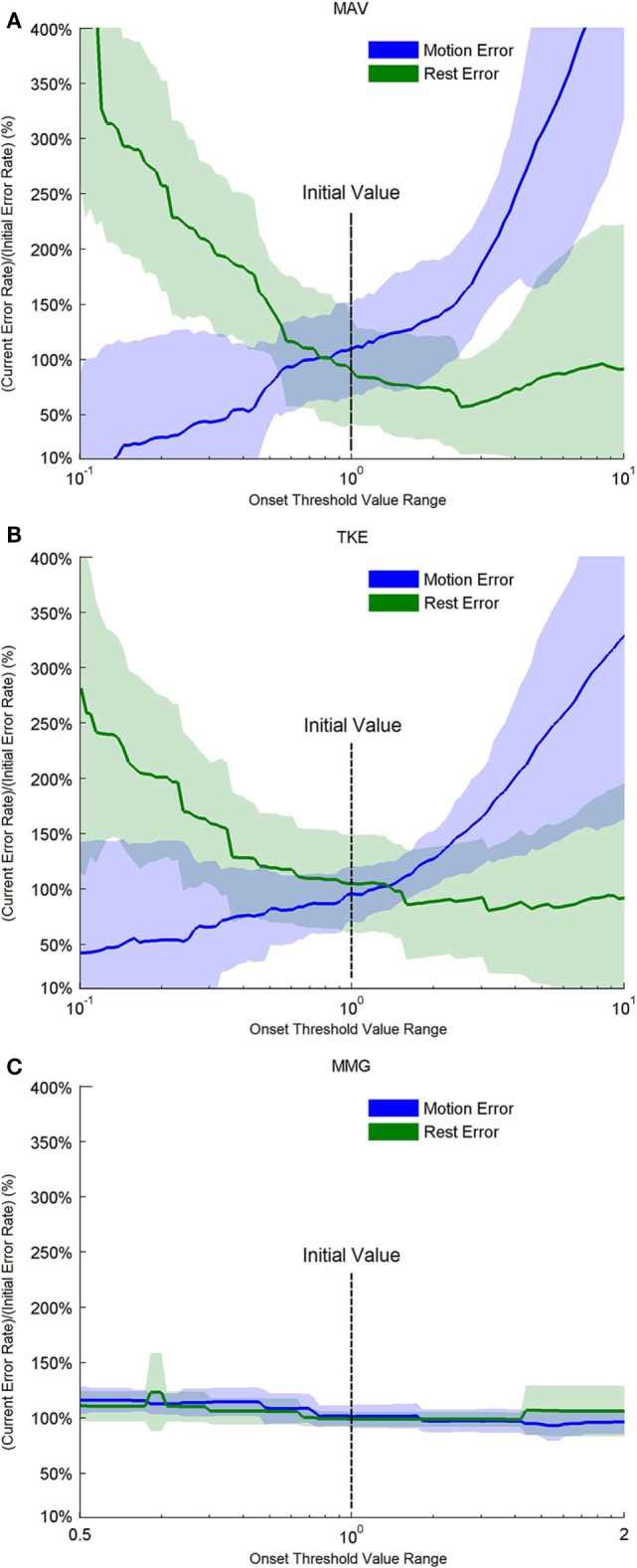
Performance based on motion and rest errors for the motion onset detectors over a range of threshold values. For **(A)** mean absolute value (MAV) and **(B)** Teager–Kaiser energy operator (TKE), the threshold values were adjusted from 0.1 to 10 times of their initial threshold values, respectively, as shown on the center of the horizontal axis. For **(C)** MMG, the threshold values were adjusted between 0.5 and 2 times of the initial threshold value.

Figure 10 shows the performance of MAV, TKE, and MMG-based thresholding schemes over a range of onset threshold values. Both MAV (Figure 10A) and TKE (Figure 10B) showed an increase of motion errors and a decrease of rest errors, as their respective onset threshold values increased from 0.1 to 10.0 times of the initial values, as calculated in Section Sensitivity Analysis of Motion Onset Detectors. This indicates that a tradeoff exists between the motion error and the rest error, while the initial value lies within the optimal range where both error types could be minimized. In addition, the slopes of both error types were smoother for TKE than those for MAV, which implies that TKE is less sensitive to the threshold value change than MAV. In addition, MMG (Figure 10C) did not show an obvious change in performance when the onset threshold value varied from half to twice of the initial value, indicating that MMG has the least sensitivity to the threshold value change among all the tested motion onset detectors.

## Discussion

### Interpretation of the Online Task Results

The results of this study have shown that the proposed postprocessing strategy could help stabilize EMG classification outputs and potentially improve the robustness of myoelectrically controlled prostheses. The performance of the strategy using each of the three different motion onset detectors namely MAV, TKE, and MMG, was evaluated, respectively. In the online testing session, as reflected by a number of metrics, the proposed strategy consistently suppressed the erroneous motion outputs in all the subjects especially during the rest and constant contraction phases, in comparison with either the original EMG classification outputs (OCO) or using a 3MV strategy. The results thus indicate that the proposed strategy could effectively improve the robustness of EMG-PR-based prosthetic control.

In this study, the offline classification error rate was relatively low across all subjects, averaged at no more than 7.8% for every motion class. However, nearly all the error metrics for evaluation of the online performance of OCO had much higher values than 7.8% (except for RER). As previous studies have demonstrated as well, the offline performance of myoelectric control does not directly reflect the online performance (Ortiz-Catalan et al., 2015). And in the online task adopted by this study, we suspect that the discrepancy stems from several factors as follows. On the one hand, any deviation from the designated sequence would be counted as an error by the proposed metrics. Thus, the errors also include those induced by the response delay of the subjects, apart from the classification errors of the EMG-PR scheme. On the other hand, the misclassifications that occur during the motion onsets and offsets as well as the resting phase between two consecutive motions, may be omitted in the offline test. This usually happens when the subject starts a contraction before the prompt appears and holds through the recording period, while no signal is acquired during the resting phase. Furthermore, the multiple types of erroneous outputs can influence the real-time task completion process to different degrees; for example, active errors are more detrimental than non-active errors, as previously mentioned. As a result, a single metric may not be sufficient to quantify the online performance of myoelectric control. Therefore, three overall metrics (TER, MER, and RER) plus another three motion-specific metrics (MSTER, MSAER, and MSSR) were adopted in this study for online performance evaluation.

It should be noted that the values of the overall metrics (TER, MER, and RER), which corresponds to the error rates during different muscle contraction periods, are higher than the offline classification error rates for the OCO. Due to the response delays of both the subjects and the control scheme, the timing differences between the predesigned sequence and the actual motion sequence performed by the subject in real-time are hardly evitable, which are also counted as errors even if they are not misclassifications. Therefore, the overall metrics assess not only the classification accuracy of the EMG-PR scheme, but reflect the actual performance of the online task by the subject with the control system as a whole. The results have shown that, the proposed postprocessing strategy could reduce both rest and motion errors by approximately 60–80 and 30–50%, respectively, and their standard deviations were largely reduced as well, compared with both the OCO and the 3MV (Figure 6) scenarios. Hence the unwanted motion outputs during both rest and contraction phases could be effectively and consistently suppressed by the strategy.

In addition, the motion specific metrics were used to assess the control performance from three aspects: total error rate (MSTER), active error rate (MSAER), and switching rate (MSSR). Both the MSTER and the MSAER, as well as their standard deviations across subjects, were significantly reduced with the proposed strategy (Figures 8A,B), indicating its ability to suppress erroneous outputs during constant contractions. Moreover, the strategy nearly eliminated the unwanted motion output changes during constant contractions, as reflected by the MSSR in Figure 8C. Thus, the proposed postprocessing strategy proved its efficacy and consistency in stabilizing the motion outputs, which would lead to a higher robustness and thus an improved online performance of prosthetic control.

### Analysis of the Postprocessing Strategy

The proposed strategy works by using a threshold to detect the motion onset and then locking the EMG classification outputs until the arrival of the motion offset. The current commercial prostheses that adopt either an on/off approach or a finite-state-machine approach are also activated by a threshold; however, using the same threshold to map muscle activities to motion classes severely limits the number of DoFs these prostheses are able to control (Farina et al., 2014). In contrast, the thresholding scheme in the proposed strategy serves as a postprocessing step that is added to the EMG-PR-based prosthetic control system, thus retaining its intuitiveness and the number of DoFs. We observed that a major contribution to the improvement of the overall performance was from the constant outputs during the *Locking stage*. These outputs were determined as the *decision output* by the *Decision stage*, where a multipoint majority vote is applied to the EMG classification outputs in the first several windows after the motion onset. The strategy assumes that the *decision output* is the motion class that the subject intends to perform throughout the contraction period. In this way, almost any interference such as muscle fatigue during the contraction that may cause misclassifications, could be blocked out.

However, this study only tested muscle contractions around 2 s, thus it remains to be seen if the strategy could retain its efficacy under even longer contractions, but presumably so long as the signal is above the offset of the selected motion onset detector, the motion output would be stable. In addition, if a motion class has a relatively low accuracy from offline classification, the *decision output* is likely to be incorrect after the majority vote (Englehart and Hudgins, 2003). Therefore, the performance of the proposed strategy depends on the original EMG classification accuracy, and the efficacy of the strategy is based on the precondition that the offline classification accuracy of any motion class is at least acceptable. Also, the proposed strategy’s performance would be affected by electrode shift in practical use of myoelectric control systems. Previously proposed methods that addressed this issue may be incorporated into the scheme of the strategy to maintain its efficacy under these interferences (Young et al., 2011). Like other postprocessing methods, one limitation of the proposed strategy is the delay produced at contraction onsets and offsets. The length of delay depends on the motion onset detector implemented in the thresholding scheme, which will be discussed in the next section. In addition, the number of windows used to determine the *decision output* through a multipoint majority vote was arbitrarily set as six in this study, whereas an optimal value has yet to be determined. Moreover, the current strategy scheme does not support simultaneous control where multiple motion classes are performed simultaneously or even with time-varying combinations. Recent studies have demonstrated the possibility of decoding complex motions or free-arm movements from EMG such as drawing figures, through a dynamic recurrent neural network approach (Dipietro et al., 2003; Cheron et al., 2007). In particular, similar to motion onset detection in this study, EMG burst detection has been used to predict leg movements from shoulder activity and was achieved by a dynamic recurrent neural network in a later study (Cheron et al., 2007), which could even generalize and predict movements beyond the training set. However, these methods are based on analyzing the whole EMG segments during muscle contractions, which might be difficult to directly implement in prosthetic applications where real-time motion identification is required. Nonetheless, they offer a further exploitation of the EMG signal and could pave the way for the development of more intelligent prosthetic systems. The proposed strategy in its current form may not be directly applicable in those control schemes. One possible solution to this limitation is using probability-based and history-dependent algorithms to adaptively adjust the threshold values, and modifying the thresholding scheme to allow multiple DoFs of motion outputs at the same time, which remains to be explored in the future.

### Comparison among Motion Onset Detectors

The effectiveness of the proposed strategy also relies on the performance of the motion onset detector, especially the stability (or smoothness) and the responsiveness (or correlation with the timing of motion onset and offset), which are the metrics used in previous studies (Wininger et al., 2008; Connan et al., 2016). In this study, although all the three adopted motion onset detectors (MAV, TKE, and MMG) achieved similar improvements in the online performance, the derivatives of EMG (MAV and TKE) failed to work for two subjects. We did not observe distinct waveforms during certain motion classes performed by these two subjects, and thus the threshold values could not be determined, which was possibly caused by the presence of noise in the EMG signal. In contrast, the MMG-based scheme effectively worked across all the subjects involved in this study, indicating that MMG may remain applicable even when the motion EMG signals are weak. Besides, Figure 9 indicates that the MMG signal has a much higher smoothness than both MAV and TKE, which has been corroborated by a recent study as well (Connan et al., 2016). For further comparison, we assessed the stability of MAV, TKE, and MMG by reconstructing their respective performance over a range of threshold values. As shown in Figure 10, when the threshold value varied, MMG showed merely slight perturbation in performance as reflected by the motion and rest errors; however, the other two motion onset detectors were highly subject to changes in the threshold value, especially MAV. Therefore, MMG is the most stable one among all the three motion onset detectors in terms of sensitivity to threshold value changes, and when EMG electrodes are the only available sensors, TKE would be more appropriate than MAV as a motion onset detector if stability is of high priority.

On the other hand, MMG is not as responsive as the derivatives of EMG, namely MAV and TKE, for motion onset detection. In fact, compared with MAV and TKE, MMG resulted in a relatively larger delay at both the beginning and the end of a contraction, thus the offset threshold value was set much higher than the onset threshold for MMG. As a result, active contractions often have to start from rest or nearly rest, which impedes rapid switches between consecutive active contractions. Besides, we found that the sensitivity of MMG in motion onset detection is related to the subcutaneous fat interfering between skin and muscle. For people with dense subcutaneous fat, the shape change of the muscle may be dampened, which might reduce the sensitivity of MMG and thus motion onset detection. For slim people whose arm muscles are small, the MMG sensor (FSR) is more likely to shift on the skin surface, which might render the MMG signal unstable and impede motion onset detection. One potential solution to this issue is to use other types of MMG sensors which are more sensitive, such as microphones (Orizio, 2004), or to combine different MMG sensors to increase the sensitivity. Another limitation of MMG is its potential influence on the EMG due to a rigid mechanical discontinuity on the muscle surface introduced by the MMG sensors (Orizio, 2004). Although this effect was quite slight according to the classification results of this study, the MMG sensors might shift over time, leading to a lower reliability of the proposed strategy. In contrast, MAV and TKE as derivatives of EMG, requires no additional sensor apart from the EMG electrodes. Besides, MAV and TKE can drop quickly to the baseline during the motion offset, enabling a much faster transition between two consecutive contractions than MMG.

In general, for the currently proposed strategy scheme, TKE would be the most applicable among the three motion onset detectors, considering the improvement in performance and the robustness, as well as the need for other sensor types. On the other hand, the MMG-based scheme has produced the most significant improvement in the control performance, and it is the most robust against threshold value changes in this study. In addition, MMG is expected to be more insensitive to the long-term change in muscle contractions as a result of muscle fatigue (Wan et al., 2010). Therefore, MMG is still a promising motion onset detector and its use in myoelectric control would benefit the chronic robustness of limb prostheses. We speculate that a combination of MMG and derivatives of EMG could utilize the advantages of both types of motion onset detectors. Moreover, a recently proposed EMG amplitude estimator based on Bayesian filtering (Hofmann et al., 2016) has proved smoother and more responsive to sudden changes in the EMG signal than MAV. Further exploration of such alternative motion onset detectors would add to the improvement of the proposed postprocessing strategy in myoelectric control.

### Outlook

We have observed obvious improvements in the online robustness of EMG-PR-based control through the proposed postprocessing strategy. It was worthy of note that the online task was based on a virtual environment rather than a real prosthesis. It needs to further investigate whether the proposed strategy would work equally well on amputees when they are wearing actual prostheses. In practical use, additional noise or interference is inevitable, which would affect the reliability of motion onset detection. Furthermore, amputees generally have lower motion classification accuracy than able-bodied people. In particular, the electrode configuration (on the forearm) in this study was intended to simulate the use of the prosthetic device by transradial amputees, whereas for transhumeral amputees, the electrodes are usually placed on their shoulders, and EMG classification for multiple motion classes can be even more difficult. These factors might influence the accuracy of the *decision output*. In this study, we have made a preliminary investigation into the efficacy of the proposed strategy on two transradial amputees using the same online testing scheme, and the improvements in their performance were comparable to those in the able-bodied subjects. Nevertheless, an additional study on the effectiveness of the strategy involving more amputees wearing real prostheses will be carried out in the future.

## Ethics Statement

The protocol of this study was approved by the ethics committee of Institutional Review Board of Shenzhen Institutes of Advanced Technology, Chinese Academy of Sciences, China.

## Author Contributions

XZ helped in conceiving the study concept, collecting, analyzing, and interpreting the data; drafting manuscript. XL, OS, and ZH helped in interpreting the data and critically revising the manuscript. PF and GL helped in conceiving the study concept, obtaining funding, and supervising the study. All authors read and approved the final manuscript.

## Conflict of Interest Statement

The authors declare that the research was conducted in the absence of any commercial or financial relationships that could be construed as a potential conflict of interest.
